# Diversity, Methane Oxidation Activity, and Metabolic Potential of Microbial Communities in Terrestrial Mud Volcanos of the Taman Peninsula

**DOI:** 10.3390/microorganisms12071349

**Published:** 2024-07-01

**Authors:** Alexander I. Slobodkin, Igor I. Rusanov, Galina B. Slobodkina, Aleksandra R. Stroeva, Nikolay A. Chernyh, Nikolai V. Pimenov, Alexander Y. Merkel

**Affiliations:** 1 Winogradsky Institute of Microbiology, Research Center of Biotechnology, Russian Academy of Sciences, Leninskiy Prospect, 33, Bld. 2, 119071 Moscow, Russia; rusanov_igor@mail.ru (I.I.R.); gslobodkina@mail.ru (G.B.S.); chernyh3@yandex.com (N.A.C.); npimenov@mail.ru (N.V.P.); alexandrmerkel@gmail.com (A.Y.M.); 2 Lomonosov Moscow State University, 119234 Moscow, Russia; a.r.stroeva@yandex.ru

**Keywords:** mud volcano, methane, AOM, ANME, methanotroph, archaea, *Methyloprofundus*, diversity, metagenome, MAG

## Abstract

Microbial communities of terrestrial mud volcanoes are involved in aerobic and anaerobic methane oxidation, but the biological mechanisms of these processes are still understudied. We have investigated the taxonomic composition, rates of methane oxidation, and metabolic potential of microbial communities in five mud volcanoes of the Taman Peninsula, Russia. Methane oxidation rates measured by the radiotracer technique varied from 2.0 to 460 nmol CH_4_ cm^−3^ day^−1^ in different mud samples. This is the first measurement of high activity of microbial methane oxidation in terrestrial mud volcanos. 16S rRNA gene amplicon sequencing has shown that *Bacteria* accounted for 65–99% of prokaryotic diversity in all samples. The most abundant phyla were *Pseudomonadota*, *Desulfobacterota*, and *Halobacterota*. A total of 32 prokaryotic genera, which include methanotrophs, sulfur or iron reducers, and facultative anaerobes with broad metabolic capabilities, were detected in relative abundance >5%. The most highly represented genus of aerobic methanotrophs was *Methyloprofundus* reaching 36%. The most numerous group of anaerobic methanotrophs was ANME-2a-b (*Ca.* Methanocomedenaceae), identified in 60% of the samples and attaining relative abundance of 54%. The analysis of the metagenome-assembled genomes of a community with high methane oxidation rate indicates the importance of CO_2_ fixation, Fe(III) and nitrate reduction, and sulfide oxidation. This study expands current knowledge on the occurrence, distribution, and activity of microorganisms associated with methane cycle in terrestrial mud volcanoes.

## 1. Introduction

Mud volcanism is a geological phenomenon of great importance for hydrocarbon exploration, seismicity, and the atmospheric budget of methane [1]. Terrestrial mud volcanoes (TMVs) are the structures located on the Earth’s surface but geologically connected to deep hydrocarbon-bearing sedimentary basins and, thus, to the subsurface biosphere [2]. Morphological structures of TMVs are diverse and include mud pools, salsa lakes, and gryphons. The breccia, liquid, and gas emissions from TMVs contain different inorganic and organic compounds, which can be used as electron donors and acceptors in microbial metabolism. Unlike marine mud volcanoes, where the main electron acceptor is sulfate, the main terminal electron acceptors in TMVs are probably iron minerals [3,4].

Microbial communities inhabiting TMVs have been studied by cultivation and molecular approaches. Aerobic and anaerobic microorganisms were isolated from TMVs located in different geographical regions [5,6,7,8,9,10,11]. TMVs in Azerbaijan, China, Iran, Italy, Japan, Romania, Russia, Taiwan, and Trinidad were inspected in relation to microbial community composition by 16S rRNA gene amplicon sequencing [5,12,13,14,15,16,17,18,19,20,21,22,23,24]. Metagenomic analyses of the genomes and functional genes were also applied to explore the diversity and metabolic potential of prokaryotes thriving in TMVs [3,25,26]. Overall, the results obtained point to an active methane, sulfur, and iron cycling in the studied ecosystems. However, most of these studies focus on a single environmental site, and comparisons of multiple TMVs located in a given geographic region are rare.

Methane oxidation (MO) in mud volcanoes is performed by aerobic methanotrophic bacteria and anaerobic methanotrophic archaea (ANME). Molecular surveys show the presence in TMVs of aerobic methylotrophs of the genera *Methylobacter*, *Methylomicrobium*, *Methylomonas*, *Methylophaga*, *Methyloprofundus*, and *Methylosoma* [4,12,23,26]. Various groups of ANME (ANME-1, ANME-2a-b, *Ca.* Methanoperedenaceae, ANME-3) have been detected in TMVs worldwide [23,24,26].

Anaerobic methane oxidation (AOM) is a globally important process that prevents the release of a significant portion of methane into the atmosphere [27]. The biological mechanisms of AOM are still insufficiently studied, but it is generally assumed that syntrophic interactions between ANME and partner bacteria play a pivotal role. ANME can couple methane oxidation with the reduction of terminal electron acceptors either alone, or in concert with a bacterial partner via extracellular electron transfer. AOM dependent on the reduction of sulfate, nitrate, nitrite, Fe(III), and Mn(IV) compounds has been described [28,29,30,31,32,33,34]. The metabolism of bacteria, which participate in AOM, is poorly characterized; most of these microorganisms are still not culturable. In sulfate-reducing AOM consortia, ANME are usually associated with bacteria belonging to different clades of the phylum *Desulfobacterota* [35,36]. Genomes of these bacteria always contain numerous multiheme *c*-type cytochromes [37,38,39]. The involvement of *Desulfobacterota* in Fe(III)-dependent AOM in TMVs has also been proposed [4].

The rate of AOM is a significant factor that determines methane emissions into the atmosphere. To quantify AOM, environmental samples are incubated with radioactive tracers, such as ^14^CH_4_. Values of AOM rates vary from 0.0001 nmol cm^−3^ day^−1^ for lake sediments to 10,000 nmol cm^−3^ day^−1^ for Black Sea microbial reefs. Most marine mud volcanoes exhibit high rates of microbial methane oxidation (10–1500 nmol cm^−3^ day^−1^) compared to other methane-containing environments [27,40]. However, TMVs are markedly different from marine mud volcanoes due to the absence of sulfate as a potential electron acceptor for AOM. *In situ* MO activity in TMVs has never been measured. 

The Kerch–Taman mud volcanic province is one of the most active areas of mud volcanism in Eurasia. More than 50 TMVs are located on the Taman peninsula (the Krasnodar Krai, Russia). The total thickness of the sedimentary strata of the peninsula is 10 km; the thickness of the Oligocene Neogene deposits is 7 km, with the Maikop series playing a dominant role. Hydrochemical (Li–Na and Li–Mg) geothermometers suggest that TMV waters in this area formed at depths ranging from 1 to 4.5 km [41].

For a complete understanding of the functioning of a complex microbial community, the most productive approach is a combination of two approaches: a cultivation approach based on the isolation of pure cultures and a cultivation-independent approach based on the use of molecular methods. Several new taxa of anaerobic bacteria were isolated and described from Taman TMVs [42,43,44,45,46]. Physiological studies of these microorganisms suggested the functioning of sulfur and iron biogeochemical cycles, but none of these isolates were highly represented in the community based on 16S rRNA gene profiling or metagenomic data. On the other hand, microbiological studies of the Taman TMVs via molecular methods have only been carried out for two microbial communities [4,26], and it is unclear how these limited data reflect the common features of the TMVs of peninsula.

The main objective of this study was to obtain information on biological mechanisms of methane oxidation and determine the dominant taxonomic groups of methanotrophic bacteria and archaea in Taman TMVs. In this paper, we report the taxonomic composition and methane oxidation rates of 17 microbial communities from five TMVs of the Taman peninsula. We compared prokaryotic diversity and revealed the common taxa of the different taxonomic ranks present in all TMVs and identified the major methanotrophic players of aerobic and anaerobic methane oxidation at the genus level. We obtained 29 high-quality metagenome-assembled genomes (MAGs) from the community with high AOM rate and assessed their metabolic capabilities, allowing us to obtain conclusions on possible physiological groups of microorganisms and electron donors and acceptors involved in methane oxidation. We also for the first time established the ranges and maximal values of methane oxidation activities in terrestrial mud volcanoes by the radiotracer technique. 

## 2. Materials and Methods

### 2.1. Sampling Site Description, Sample Collection, and Chemical Analysis

Samples were collected in June 2022, from the fields of five mud volcanoes located in the Taman peninsula, Krasnodar Krai, Russia. Samples were taken at a depth of 15–20 cm below the surface, placed into sterile 50 mL bottles, and transported to the laboratory where they were prepared for molecular and geochemical studies. A description of the samples is given in the Section 3.

Temperature and pH of the samples were measured at the point of collection using a digital pH meter equipped with a thermocouple (AZ Instrument, Moscow, Russia). The concentrations of chloride, sulfate, and nitrate were analyzed in water extracts by HPLC with a Stayer ion chromatograph (Aquilon, Moscow, Russia) with an IonPack AS4-ASC column (Dionex, Sunnyvale, CA, USA) and conductivity detector; the eluent was bicarbonate (1.36 mM)/carbonate (1.44 mM), and the flow rate was 1.5 mL min^−1^.

### 2.2. Methane Content and Rates of Microbial Methane Oxidation

The activity of processes of microbial oxidation of methane in the TMVs was determined by the radioisotope method. Methane concentrations were measured by the phase-equilibrium degassing method on a gas chromatograph, Kristall-2000-M (Chromatec, Yoshkar-Ola, Russia), equipped with a flame ionization detector. The measurement error did not exceed ±5%. The rate of methane oxidation (MO) was determined by the radioisotope method with ^14^C-methane ([^14^C]-methane, specific activity 1.16 GBq/mmol, JSC Isotope, Russia) dissolved in degassed distilled water. Undisturbed sediments in amounts of 2.0 cm^3^ were taken with plastic syringes (total volume 5 cm^3^) with a rubber piston and a cut edge and hermetically sealed with a butyl rubber stopper. For the variant with the addition of air, 2.0 cm^3^ of the gas phase was left. Then, 0.2 mL of labeled ^14^C-methane (1 µCi per sample) was added with a tuberculin syringe, piercing the rubber stopper with a needle in the center and evenly distributing the substrate along the length of the syringe with the sediment. Samples fixed with 1 mL of 0.5 N KOH solution before adding the labeled substrate served as controls. After adding labeled methane, sediment samples were incubated for 1 day at a temperature close to the *in situ* temperature. After incubation, the samples were fixed and transported to a stationary laboratory for further determination of the products of microbial oxidation and transformation of C-methane into carbon dioxide (CO_2_), into microbial biomass (BM), and into dissolved organic carbon (DOC) in dissolved organic matter (DOM). Sample processing and calculations of the intensity of methane oxidation were carried out according to a previously described method [47]. Radioactivity (^14^C) of the products of the microbial process of MO was measured on a TRI-CarbTR 2400 liquid scintillation counter (Packard, Conroe, TX, USA).

### 2.3. DNA Extraction and 16S rRNA Gene Amplicon and Metagenome Library Preparation, Sequencing, and Analysis

DNA from mud samples was isolated using the FastDNA Spin Kit for Soil according to the manufacturer’s protocol (MP Biomedicals, Santa Ana, CA, USA). Preparation, sequencing, and analysis of the V3-V4 region of the 16S rRNA amplicon libraries were performed as previously described [26]. qPCR-based total prokaryote quantification was performed as previously described [48].

Preparation and sequencing of a shotgun metagenome library were performed in Laboratory “Genomed” Ltd., Moscow, Russia, using the MGIEasy Fast PCR-FREE FS Library Prep Set (MGI, China) according to the manufacturer’s protocol and the DNBSEQ-G400 system (MGI, China) with the reagent kit, which can read 150 nucleotides from each end. Raw read processing, contig assembling, binning, bin refinement, coverage calculation, bin completeness and contamination evaluation were performed as previously described [4]. Taxonomic positions were assigned to each bin according to the Genome Taxonomy Database (GTDB) classification as described earlier [4].

Metagenome-assembled genomes (MAGs) were submitted for gene calling and annotations through the RAST [49]. Genes encoding metabolic functions were queried in RASTtk using Blast search. A list of functional genes queried in this study can be found in Supplementary Table S1. All the sequencing data are deposited in NCBI BioProject PRJNA940400. The MAGs Accession Numbers are JARGFH000000000—JARGGJ000000000.

## 3. Results and Discussion

### 3.1. Geochemical Characteristics of the Studied Sites

Seventeen samples of mud volcanic fluids and sediments were taken from mud pools, gryphons, and salsa lakes located on the fields of five mud volcanoes: Gnilaya Gora (45.251 N, 37.436 E), Gladkovsky (45.005 N, 37.724 E), Kuchugursky (45.432 N, 36.922 E), Semigorsky (44.901 N, 37.597 E), and Shugo (45.070 N, 37.611 E). The main geochemical parameters that control the functioning of microbial communities were determined: temperature, pH, E_h_, salinity, methane content, concentrations of chloride, sulfate, nitrate, and phosphate (Table 1). All samples had a temperature close to ambient temperature (about 23 °C) and a neutral or alkaline pH (from 6.72 to 9.10). The salinity of the samples varied from 9 to 30 g L^−1^, E_h_ from +15 to −348 mV, methane content from 3 to 917 mM, chloride concentration from 5.7 to 412 mM, and sulfate concentration from 0 to 63 mM. None of the samples had significant concentrations of nitrate and phosphate (>1 mM).

### 3.2. Microbial Methane Oxidation

**Figure 1:**
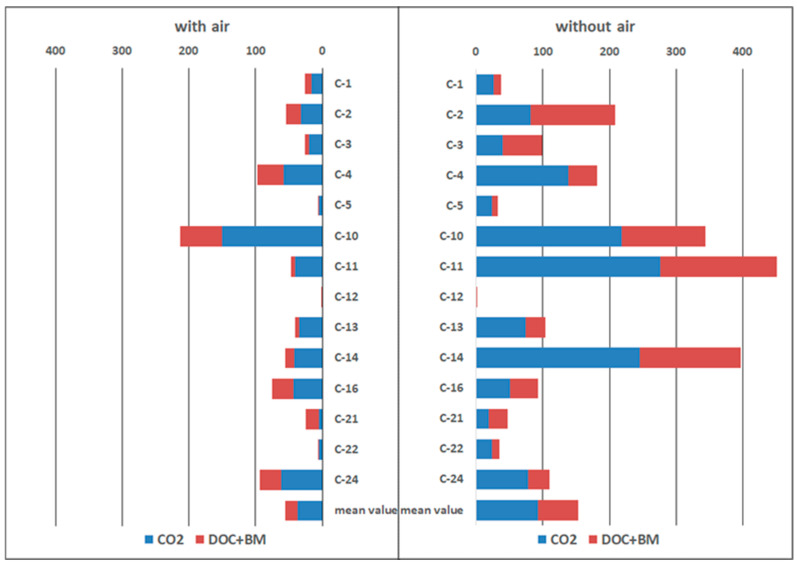
Rates of microbial methane oxidation (nmol CH_4_ cm^−3^ day^−1^) and distribution of carbon from methane into fractions.

The potential *in situ* rates of microbial methane oxidation (MO) were measured directly in the original samples by the radiotracer technique (Figure 1). In order to evaluate the effect of oxygen on methane oxidation, the samples were incubated in parallel in the presence and absence of atmospheric air in the gas phase. Air oxygen is supposed to inhibit the anaerobic oxidation of methane and stimulate the aerobic oxidation. The experimental results showed high MO values, both in aerobic (maximum of 213 nmol CH_4_ cm^−3^ day^−1^, with an average value of 50 nmol CH_4_ cm^−3^ day^−1^) and anaerobic conditions, reaching values of 400–460 nmol CH_4_ cm^−3^ day^−1^ (average value of 150 nmol CH_4_ cm^−3^ day^−1^). These values are several times higher than those of MO activity in the surface sediments of the underwater mud volcano Haakon Mosby (72 degrees N) and in the gas seepage fields of the Vestnesa Ridge, where the maximum values of microbial methane oxidation reached 70 nmol CH_4_ cm^−3^ day^−1^ [50]. The highest MO rates were observed in the samples from the TMV Gladkovsky. Moreover, the MO rates under anaerobic conditions significantly (in most cases by several times) exceeded the MO rates in the presence of air. Under aerobic conditions, methane consumption ranged from 2 to 39% of the total methane injected into the sample, whereas under anaerobic conditions, 10 to 98% of the total methane injected into the sample was consumed. These results indicate the activity of methanotrophic anaerobic microorganisms in the studied TMVs.

During methane oxidation, the carbon from methane is transformed not only into carbon dioxide but is also included in biomass as well as in various low-molecular-weight extracellular dissolved organic compounds (DOC), which can serve as a carbon source for heterotrophic microorganisms. In our experiments, the average proportion of the carbon from methane transformed into carbon dioxide was about 64% for all investigated TMVs (Figure 1). The carbon from methane in DOC was on average 35%, and only 1–2% of the methane carbon was assimilated into the biomass. These data indicate the important role of exometabolites in methane oxidation.

### 3.3. Microbial Community Abundance and Composition

Microbial communities of the studied sites differed significantly in the prokaryote total number estimated by 16S rRNA gene-based qPCR. The highest values of total prokaryote abundance were found in site C20 of the Semigorsky volcano (4.6 × 10^9^ 16S rRNA gene copies (g.c.) calculated per 1 mL of mud pool sediment, standard deviation (SD) 7.3%), site C05 of the Gnilaya Gora volcano (2.9 × 10^9^ 16S rRNA g.c. mL^−1^ of mud pool sediment, SD 12.3%), and site C14 of the Gladkovsky volcano (2.9 × 10^9^ 16S rRNA g.c. mL^−1^ of mud pool sediment, SD 9.9%). The lowest density of microbial communities was found in site C02 of the Gnilaya Gora volcano (1.2 × 10^5^ 16S rRNA g.c. mL^−1^ of gryphon mud, SD 2.4%), site C10 of the Gladkovsky volcano (3.6 × 10^5^ 16S rRNA g.c. mL^−1^ of mud pool sediment, SD 5.3%), and site C24 of the Shugo volcano (3.8 × 10^5^ 16S rRNA g.c. mL^−1^ of gryphon mud, SD 8.1%). All values are shown in Supplementary Table S2.

Using high-throughput sequencing of 16S rRNA gene amplicons, the phylogenetic composition of 17 microbial communities from five TMVs was determined. A total of 137,673 sequences of the 16S rRNA gene V3-V4 region were taken for analysis after all the initial steps of data processing. These sequences were merged into 967 amplicon sequence variants (ASVs). Alpha diversity metrics such as Chao1 richness (the predicted total number of phylotypes in a community) and the Shannon index (index of the complexity of the internal structure of a community) were calculated after normalization of the data via rarefying. The highest and the lowest values of these indexes were found in sites of the Gladkovsky mud volcano. Sites C12 and C13 had Chao1 richnesses of 121 and 142 and Shannon indices of 4.15 and 4.28, respectively, whereas sites C10 and C14 had Chao1 richnesses of 41 and 60 and Shannon indices of 2.25 and 2.59, respectively. All values are shown in Supplementary Table S3.

**Figure 2:**
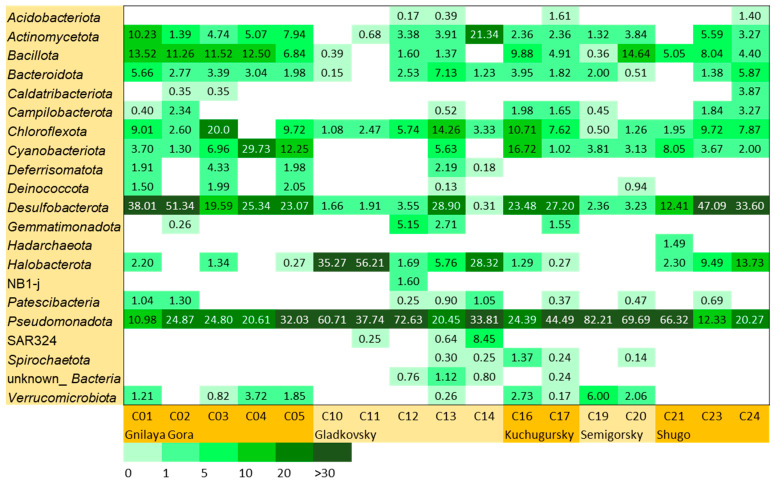
The relative abundance of prokaryotic phyla (Silva and GTDB taxonomy), which is more than 1% in at least one of the samples.

Bacteria dominated almost all the studied samples, accounting for 65–99%, with the exception of one sample (C11), where archaea of the phylum *Halobacterota* constituted 56% of all the prokaryotic sequences obtained. Twenty-one phyla (Silva and GTDB taxonomy) of *Bacteria* were found in relative abundance of more than 1% in at least one sample (Figure 2). Eight phyla made up a significant proportion of the community in most samples: *Pseudomonadota* (11–82%), *Halobacterota* (1–56%) *Desulfobacterota* (2–51%), *Cyanobacteriota* (1–30%), *Actinomycetota* (1–21%), *Chloroflexota* (1–20%), *Bacillota* (1–15%), and *Bacteroidota* (1–7%). In general, these data are consistent with those obtained for other TMV microbial communities on the Eurasian continent, suggesting that, due to the lack of fluid exchange, TMVs represent a patchy habitat populated by microorganisms highly adapted to local environmental conditions [20]. Taman TMVs also contain a high percentage of *Halobacterota*, *Desulfobacterota*, and *Pseudomonadota*, which is typical for seafloor methane seeps [51].

**Figure 3:**
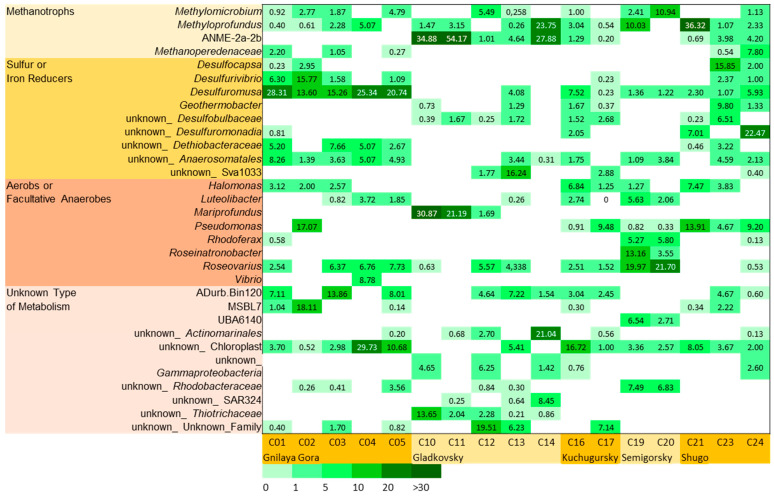
Relative abundance of prokaryotic genera (Silva and GTDB taxonomy), which is more than 5% in at least one of the samples. Taxa are grouped based on the metabolic properties of phylogenetic relatives.

Thirty-one prokaryotic genera were detected in relative abundance of more than 5% in at least one sample (Figure 3). Based on the metabolic capabilities of the cultivated representative of these genera, the identified microorganisms can be divided into four physiological groups: (1) methanotrophs; (2) sulfur or iron reducers; (3) aerobes or facultative anaerobes; (4) bacteria with an unknown type of metabolism. 

Aerobic methanotrophic bacteria were present in all the samples. *Methyloprofundus* was the most highly represented genus of methanotrophs found in all studied TMVs, with relative abundance reaching 36%. *Methyloprofundus* spp. have been detected by molecular methods in many sites all around the world, mainly in marine sediments, while the cultivated members of the genus are limited to a single species [52,53]. Methanotrophic aerobes of the genus *Methylomicrobium* also constituted a significant part of some studied microbial communities.

**Figure 4:**
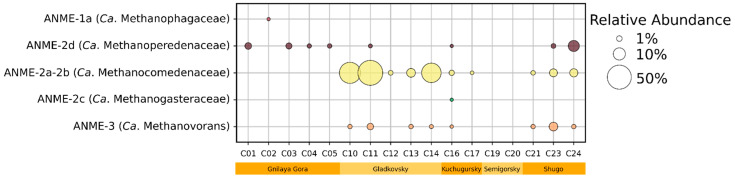
The relative abundance of different ANME groups in the studied samples.

Anaerobic methanotrophic archaea (ANME) belonging to different phylogenetic groups [54] were detected in 15 out of 17 samples (Figure 4). The relative abundance of ANME varies from 0 to 56.2% in all prokaryotic sequences. The most widely represented and numerous group was ANME-2a-b (*Ca.* Methanocomedenaceae), identified in 10 samples. Other detected ANME groups were present in smaller proportions and included the following: ANME-3 (*Ca.* Methanovorans), ANME-2d (*Ca.* Methanoperedenaceae), ANME-1a (*Ca.* Methanophagaceae), and ANME-2c (*Ca.* Methanogasteraceae). The highest relative abundance of ANME was found in the mud pools of the TMV Gladkovsky (up to 54% ANME-2a-b).

The ability to dissimilate sulfur or iron compounds is characteristic for microorganisms of the phylum *Desulfobacterota* (Figure 3). Microbial processes performed by these bacteria can include sulfate reduction (*Desulfocapsa*, unclassified *Desulfobulbaceae*), elemental sulfur reduction (*Desulfuromusa*, *Desulfurivibrio*, *Desulfocapsa*, unclassified *Desulfuromonadia*, unclassified *Dethiobacteraceae*), sulfur disproportionation (*Desulfurivibrio*, *Desulfocapsa*). The majority of sulfur-reducing and sulfur-disproportionating genera were detected in the samples from the TMVs Gnilaya Gora and Shugo. Bacteria capable of Fe(III) reduction included *Geothermobacter*, unclassified *Desulfobulbaceae*, and *Desulfuromusa*. Recently, the capacity for iron reduction has been demonstrated for OPB41 bacteria, which were described as a new order, *Anaerosomatales* [55]. Also, the uncultured Sva1033 (*Desulfuromonadales*) bacterial group could be involved in Fe(III) reduction [56]. Possibly, iron and sulfur reducers are syntrophic partners of ANME and complete electron transfer to the terminal acceptor. 

Several identified cosmopolitan genera of *Gammaproteobacteria* with high relative abundance are known as microaerophiles or facultative anaerobes with broad metabolic capabilities. For example, except organoheterotrophic lifestyle, *Halomonas* spp. have the abilities for denitrification and heterotrophic sulfur oxidation, and some species of *Roseovarius* can grow lithoautotrophically utilizing sulfur compounds, molecular hydrogen, and nitrate [45,57]. The genus *Mariprofundus* is characterized by obligate lithoautotrophic microaerobic oxidation of Fe(II) [58]. 

### 3.4. MAGs’ General Characteristics and Phylogenetic Identification

Based on a high microbial population density (2.9 × 10^9^ 16S rRNA g.c. mL^−1^ of mud pool sediment), high ANME-2a-2b (*Ca.* Methanocomedenaceae) content, and high rate of methane oxidation, site C14 of the Gladkovsky volcano was chosen for further metagenomic study. Shotgun sequencing resulted in a ~12 Gb that was assembled in 30,910 contigs with a total length of 147 Mb (N50—9401b).

Twenty-nine MAGs with >70% completeness and <4.0% contamination were recovered from the metagenome. The MAGs statistics, including the number of contigs, genome size, the presence of 16S rRNA gene, and relative abundance calculated on the basis on coverage estimation, are shown in Table 2. 

According to a phylogenomic analysis based on GTDB 08-RS214 [59], 25 of 29 MAGs were assigned to the domain *Bacteria* and 4 were assigned to the domain *Archaea*. Almost all the MAGs belonged to currently uncultivated microorganisms of different taxonomic ranks. MAG C14-19, the second most abundant in metagenome, and MAG C14-15 represent novel phyla in the domain *Bacteria*. Three MAGs (C14-28, C14-29, and C14-12) belong to new bacterial classes: three to new orders; nine to new families; and seven to new genera. Only five MAGs could be assigned to the described genera (*Methyloprofundus*, *Brevefilum*, *Ca.* Methanovorans, *Lutibacter*, and *Sedimenticola*). A complete (>1400 bp) 16S rRNA gene was recovered in 7 of 29 MAGs. The most abundant taxa present in the metagenome were members of uncultivated c_*Acidimicrobiia* (19%), unclassified d_*Bacteria* (18%), two populations of anaerobic methanotrophic archaea of f_*Ca.* Methanocomedenaceae (14 and 12%), and aerobic methanotrophs of the genus *Methyloprofundus* (9%). The other 24 MAGs had relatively low abundance (0.3–3%).

Anaerobic methanotrophic archaea were represented by three MAGs. C14-17 and C14-08, belonging to *Ca.* Methanocomedenaceae (ANME-2a-2b), together accounted for 26% of the community. This is quite close to the assessment obtained when analyzing the V3-V4 16S rRNA gene profiles (27.9%). Phylogenetically, they appeared to be very close to the previously described MAGs from the methanotrophic enrichment culture from the Gladkovsky mud volcano [4]: ANI between C14-17 and B4-04 (GCA_023544575.1)—99.4% and between C14-08 and B4-03 (GCA_023544605.1)—96.2% (Supplementary Figure S1). The third MAG (C14-18) belonged to the *Ca.* Methanovorans (ANME-3), accounted for 0.4% of the community, and was also very close to the minor MAG from the same enrichment culture: ANI between C14-18 and B4-05 (GCA_023544565.1)—99.5% (Supplementary Figure S1).

### 3.5. Metabolic Capabilities in MAGs

**Figure 5:**
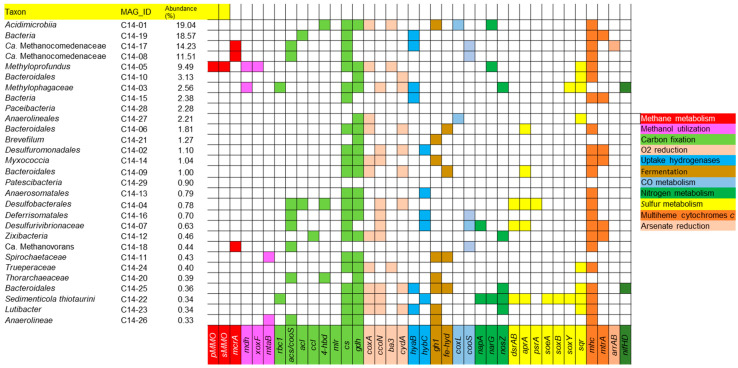
Metabolic capabilities encoded in MAGs of the sample C14 from the TMV Gladkovsky. Genes present in MAGs are shown in the bottom row. Gene abbreviations are explained in the text and listed in Supplementary Table S1.

The presence of the functional genes related to energy metabolism and carbon and nitrogen fixation in the MAGs is shown in Figure 5.

Primary production of organic matter in this microbial community may be partly due to methane oxidation. The genomes of methanotrophic archaea (*Ca.* Methanocomedenaceae, *Ca.* Methanovorans), which account for 26% of the prokaryotic sequences, contain the gene of methyl coenzyme M reductase (*mcrA*), responsible for the anaerobic oxidation of methane [60]. Genes of particulate methane monooxygenase (*pMMO*) as well as soluble methane monooxygenase (*sMMO*), performing the initial step of aerobic methane oxidation, are present in the genome of *Methyloprofundus*, which is also abundant in the studied community. Methanol, likely formed as an intermediate of methane oxidation abiotically or by methanotrophs [61,62], may be used aerobically by *Methylophagaceae* or anaerobically by *Spirochaetaceae* and *Anaerolineae*, whose genomes contain genes of methanol dehydrogenase (*mdh*) or methanol:corrinoid methyltransferase (*mtaB*). To date, the ability to utilize methanol has been found in only one bacterium isolated from a TMV: *Pelomicrobium methylotrophicum* [44].

The genes of the key enzymes involved in autotrophic CO_2_ fixation were identified in almost all MAGs, excluding two MAGs belonging to *Paceibacteria* and *Patescibacteria*. This does not mean that the majority of microorganisms in this community are autotrophs, but it does indicate the possible distribution of the pathways of carbon fixation in the studied ecosystem [63]. The genes of ribulose bisphosphate carboxylase form I (RuBisCO, *rbc1*), the main enzyme of the Calvin−Benson−Bassham cycle, were found in the MAGs of *Methylophagaceae* and *Sedimenticola thiotaurini*. The members of the genus *Sedimenticola* are facultative lithoautotrophs, and recently a new species—*S. hydrogenitrophicus*, capable of lithoautotrophic growth with molecular hydrogen or thiosulfate as electron donors and nitrate, nitrous oxide, or oxygen as electron acceptors—was isolated from the studied TMV Gladkovsky [46]. CO dehydrogenase/acetyl-CoA synthase (*acs/cooS*), the key catalytic complex of the Wood−Lyngdahl pathway (WL) was encoded in 8 out of 29 MAGs including all *Archaea* and the majority of *Desulfobacterota*. Similarly, WL predominates over other carbon assimilation pathways in AOM microbial communities of the Karabetova Gora mud volcano, also located on the Taman peninsula [26]. Marker genes of other CO_2_ fixation pathways were less common. ATP-citrate lyase (*acl*) and citryl-CoA lyase (*ccl*) (two variants of the reductive tricarboxylic acid cycle) were encoded in unknown Bacteria (MAG C14-19), *Desulfobacterales,* and *Zixibacteria*. The genes of 4-hydroxybutyryl-CoA dehydratase (*4-hbd*) (3-hydroxypropionate/4-hydroxybutyrate and dicarboxylate/4-hydroxybutyrate cycles) were present in *Acidimicrobiia*, *Desulfobacterales,* and *Thorarchaeaceae*. The genes of malonyl-CoA reductase (*mlr*, 3-hydroxypropionate bi-cycle) were not found in any MAG. Two carbon fixation pathways—the reversed oxidative tricarboxylic acid cycle and reductive glycine pathway—cannot be identified based on genomic data [64,65,66]. However, the genes of citrate synthase (*cs*) and glycine dehydrogenase (*dgh*) required for these assimilation processes were present in most of the MAGs.

Half of the MAGs contained genes of the oxygen reduction enzymatic systems: cytochrome *c* oxidase of *aa_3_*, *cbb_3_*, or *ba_3_* types or cytochrome *d* ubiquinol oxidase (*coxA*, *ccoN*, *ba3*, and *cydA* genes, respectively). These enzymes can be used for either respiration or detoxification [67], but it is not possible to distinguish between these processes based on genomic information alone. A similar situation was observed in another Taman TMV, Karabetova Gora, where 35% of the microbial community members were potential aerobes [26]. Most probably, oxygen penetrated from the atmosphere is constantly present in the upper layers of the mud. However, the MAGs of all detected *Archaea* as well as of unknown *Bacteria*, *Paceibacteria*, *Brevefilum*, *Patescibacteria*, *Anaerosomatales*, *Spirochaetaceae*, and *Anaerolineae* did not encode any cytochrome oxidases indicating the strictly anaerobic lifestyle of these microorganisms.

Genes of uptake hydrogenases (*hyaB*, *hybC*) were present in 37% of the MAGs, indicating the importance of hydrogen metabolism in the community. One of *Methanocomedenaceae*, two unknown *Bacteria* (C14-19 and C14-15), *Methylophagaceae*, *Bacteroidales*, *Sedimenticola*, and *Lutibacter* have the potential to utilize molecular hydrogen. It should be noted that the detection of genes encoding [NiFe]-hydrogenase in ANME-2 (*Methanocomedenaceae*) suggests the ability for hydrogenotrophy, which is still unknown for this group of methanotrophic archaea. Previously, uptake hydrogenases genes were found in ANME-2 only in one study: in MAGs recovered from metagenome also associated with TMVs located on the Taman peninsula [4].

Genes of periplasmic Fe-Fe hydrogenases (*fe-hyd*) or GH1 betaglucosidase (*gh1*) indicative of fermentation were present in 30% of the MAGs, including the most abundant *Acidimicrobia*. A gene of the large chain of aerobic carbon monoxide dehydrogenase (*coxL*) was found in the MAGs of *Acidimicrobia* and *Anaerolineaceae*. Genes encoding the catalytic subunit of anaerobic CO-dehydrogenase (*cooS*) were present in five MAGs. 

Anaerobic methane oxidation could be coupled to nitrate, sulfate, or Fe(III) reduction. Genes of the catalytic subunit of the respiratory nitrate reductase of the Nar type (*narG)* were identified in *Acidimicrobiia* and *Methyloprofundus* which are present in the community in high abundance. At present, the only known species of *Methyloprofundus* is *M sedimenti*. It was originally described as an aerobic methanotroph utilizing nitrate as a nitrogen source, and anaerobic growth with nitrate reduction was not tested [52]. Our genome analysis revealed that in addition to *nasAB* involved in nitrate assimilation, the genome of *M sedimenti* possesses *narGHI*, *nirK*, *nirS*, and *norBC* genes and does not contain *nosZ*. The class *Acidimicrobiia* has more than 4000 members according to GTDB. The order to which MAG C14-01 was assigned includes only uncultivated bacteria; so, their physiology and metabolism are unknown. Periplasmic nitrate reductase of the Nap type (*napA*) was encoded in *Desulfurivibrionaceae* and *Sedimenticola.* The capacity to grow with nitrate reduction is known for the representatives of these taxa [68,69]. The terminal enzyme of the complete denitrification, nitrous oxide reductase (*nosZ*), was found in five MAGs. Only MAG C14-22 (*Sedimenticola*) had both nitrate and nitrous oxide reduction genes.

The oxidative branch of the sulfur cycle was prominent in the MAGs. The key enzyme for sulfide oxidation, sulfide:quinone oxidoreductase (SQR), appears to be a ubiquitous protein present in both chemotrophic and phototrophic prokaryotes as well as in some mitochondria [70]. The presence of *sqr* was found in 8 of 29 MAGs (*Methyloprofundus*, *Bacteroidales*, *Methylophagaceae*, *Anaerolineales*, *Trueperaceae*, *Bacteroidales*, *Sedimenticola*, and *Lutibacter*). Additionally, the genes of sulfite dehydrogenase (*soeA*) and proteins of the Sox system (*soxB*, *soxY*), involved in sulfite and thiosulfate oxidation, were identified in the MAGs of *Methylophagaceae* and *Sedimenticola*. The family *Methylophagaceae* consists of the single genus *Methylophaga*, some species of which are capable of chemolithoheterotrophic growth with sulfide or thiosulfate oxidation [71,72]. All known *Sedimenticola* species are facultative-sulfur-compound-oxidizing lithoautotrophs [46]. The marker genes of sulfate reduction coding for alpha and beta subunits of dissimilatory sulfite reductase (*dsrAB*) and adenylylsulfate reductase (*aprA*) were detected in three MAGs with low abundance (*Desulfobacterales*, *Desulfurivibrionaceae*, and *Sedimenticola*). Thiosulfate/polysulfide reductase (*psrA*) was encoded only in *Desulfobacterales*.

The biochemical mechanisms of dissimilatory Fe(III) reduction are diverse, and currently, there are no universal genomic determinants of this process. However, it is well established that *c*-type cytochromes play a key role in electron transfer to the extracellular electron acceptor in many microorganisms [73]. The search for multiheme *c*-type cytochromes in the C14 metagenome revealed that 6 out of 29 MAGs contain genes of the proteins homologous to decaheme cytochrome *c* MtrA (*mtrA*), involved in iron reduction in *Shewanella oneidensis* [74]. Hence, it can be hypothesized that unknown *Bacteria* (MAG C14-19 and C14-15), *Desulfuromonadales*, *Myxococcia*, *Desulfurivibrionaceae*, and *Zixibacteria* participate in the reduction of Fe(III)-containing minerals in the studied ecosystem. 

Homologs of respiratory periplasmic arsenate reductase (*arrAB*) were encoded in the MAG of *Ca.* Methanocomedenaceae C14-17. It is the second piece of evidence of the presence of Arr in ANME-2; previously, this enzyme was detected in methanotrophic Fe(III)-reducing enrichment culture obtained from Taman TMVs [4]. As(V) reduction has been also experimentally shown for a number of cultivated bacteria isolated from Taman TMVs [9,42,43,75], but other MAGs of C14 metagenome did not contain *arrAB*. The presence of nitrogenase genes (*nifHD*) in *Methylophagaceae* and *Bacteroidales* indicated the ability to fix dinitrogen. 

In summary, five the most abundant members of the community (70% of the total sequences) contained genes indicative of aerobic and anaerobic methanotrophy, CO_2_ fixation, fermentation, Fe(III) and nitrate reduction, and sulfide oxidation. It can be assumed that two populations of *Ca.* Methanocomedenaceae couple AOM to Fe(III) reduction either directly or via syntrophic bacterial partner, which could be the unknown *Bacteria* C14-19; *Methyloprofundus* oxidizes methane aerobically in oxygenated zones of TMVs or links methane oxidation to nitrate reduction; facultative anaerobic *Acidimicrobiia* decomposes the produced organic matter.

## 4. Conclusions

Microbial communities of five mud volcanoes located at the Taman peninsula were investigated by molecular and radiotracer methods for the first time. Methane oxidation rates varied from 2.0 to 460 nmol CH_4_ cm^−3^ day^−1^ in different mud samples. MO rates under anaerobic conditions significantly exceeded the MO rates in the presence of air, indicating the activity of methanotrophic anaerobic microorganisms. Bacteria accounted for 65–99% of prokaryotic diversity in all the samples. The most abundant bacterial phyla were *Pseudomonadota*, *Desulfobacterota*, *Bacteroidota*, *Actinomycetota*, *Bacillota*, and *Chloroflexota*. Thirty-two prokaryotic genera, which include methanotrophs, sulfur or iron reducers, and facultative anaerobes with broad metabolic capabilities, were detected in relative abundance of more than 5% in at least one sample. The most highly represented genus of aerobic methanotrophs was *Methyloprofundus*. Its numbers were as high as 36% in the TMV Shugo. The most widely represented and numerous group of anaerobic methanotrophs was ANME-2a-b (*Ca.* Methanocomedenaceae), identified in 60% of samples and reaching 54% in the TMV Gladkovsky. Other detected ANME groups were present in smaller proportions and included the following: ANME-3 (*Ca.* Methanovorans), ANME-2d (*Ca.* Methanoperedenaceae), ANME-1a (*Ca.* Methanophagaceae), and ANME-2c (*Ca.* Methanogasteraceae). Twenty-nine high-quality MAGs were recovered from the metagenome of the sample with high rate of methane oxidation. Almost all the MAGs belonged to uncultivated microorganisms of different taxonomic ranks. The distribution of the functional genes between taxa points to the importance of methanotrophy, CO_2_ fixation, Fe(III) and nitrate reduction, and sulfide oxidation. Sulfate-reducing bacteria were not abundant, suggesting that in Taman TMVs, the biological mechanism of methane oxidation is not dependent on sulfate reduction but rather coupled to Fe(III) reduction. Overall, our study expands current knowledge on the occurrence, distribution, and activity of microorganisms associated with methane cycle in terrestrial mud volcanoes.

## Figures and Tables

**Table 1:** Characteristics of the studied sites.

Sample #	Volcano	Sampling Site	pH	Salinity, g L^−1^	E_h_, mV	CH_4_, mM	Cl^−^, mM	PO_4_^−^, mM	SO_4_^2−^, mM	NO_3_^−^, mM
C01	Gnilaya Gora	Gryphon	8.47	20	−295	101.1	241.1	0.00	5.79	0.00
C02	Gnilaya Gora	Gryphon	8.66	14	−175	556.7	158.8	0.07	1.07	0.00
C03	Gnilaya Gora	Salsa	9.10	20	−320	201.1	412.0	0.00	0.00	0.00
C04	Gnilaya Gora	Mud pool	8.64	12	−348	536.3	162.8	0.02	0.09	0.92
C05	Gnilaya Gora	Mud pool	8.94	20	−302	47.7	216.1	0.00	1.95	0.00
C10	Gladkovsky	Mud pool	6.75	20	−124	668.0	307.5	0.00	0.06	0.00
C11	Gladkovsky	Mud pool	6.72	20	−110	661.5	300.8	0.00	0.08	0.00
C12	Gladkovsky	Mud pool	7.07	30	15	3.3	402.2	0.00	0.00	0.00
C13	Gladkovsky	Mud pool	7.08	30	−255	105.9	393.4	0.00	0.00	0.00
C14	Gladkovsky	Mud pool	7.02	20	−217	439.1	291.2	0.00	0.00	0.00
C16	Kuchugursky	Main crater	7.43	15	−212	640.5	153.9	0.00	8.54	0.00
C17	Kuchugursky	Mud pool	6.92	20	nd	nd	147.4	0.00	63.52	0.00
C19	Semigorsky	Mud pool	8.20	10	nd	nd	5.7	0.00	0.08	0.00
C20	Semigorsky	Mud pool	8.35	9	nd	nd	37.2	0.00	0.02	0.00
C21	Shugo	Gryphon	7.23	20	−196	456.8	199.2	0.00	0.20	0.00
C23	Shugo	Gryphon	6.94	12	nd	nd	95.4	0.00	0.21	0.00
C24	Shugo	Gryphon	7.41	20	−157	917.3	193.7	0.00	0.21	0.00

nd—not determined.

**Table 2:** Overview of all genome bins >50% complete and with <5% contamination.

Bin ID	Domain	Taxon *	Abundance %	Completeness %	Contamination %	# Contigs	Genome Size, Mbp	16S rRNA Gene, bp
C14-01	B	c_*Acidimicrobiia*	19.04	100.00	2.14	181	3.91	1171
C14-19	B	d_*Bacteria*	18.57	94.78	0.84	157	3.19	1558
C14-17	A	f_*Ca.* Methanocomedenaceae	14.23	95.42	0.00	131	2.06	-
C14-08	A	f_*Ca.* Methanocomedenaceae	11.51	98.69	0.00	74	1.82	-
C14-05	B	g_*Methyloprofundus*	9.49	99.31	1.55	189	3.28	-
C14-10	B	o_*Bacteroidales*	3.13	97.85	1.90	50	3.48	1533
C14-03	B	f_*Methylophagaceae*	2.56	99.82	0.65	96	2.44	-
C14-15	B	d_*Bacteria*	2.38	96.40	0.00	255	2.72	-
C14-28	B	p_*Paceibacteria*	2.28	76.85	1.12	68	1.17	1474
C14-27	B	o_*Anaerolineales*	2.21	80.18	2.73	99	2.69	1517
C14-06	B	o_*Bacteroidales*	1.81	99.28	1.90	74	3.81	-
C14-21	B	g_*Brevefilum*	1.27	90.00	2.73	104	2.66	1522
C14-02	B	o_*Desulfuromonadales*	1.10	100.00	0.22	118	2.72	-
C14-14	B	c_*Myxococcia*	1.04	96.55	2.60	75	6.11	768
C14-09	B	o_*Bacteroidales*	1.00	98.57	1.19	119	4.61	-
C14-29	B	p_*Patescibacteria*	0.90	73.76	0.99	49	1.46	1512
C14-13	B	o_*Anaerosomatales*	0.79	96.66	3.61	52	1.98	432
C14-04	B	o_*Desulfobacterales*	0.78	99.35	2.26	269	4.94	556
C14-16	B	o_*Deferrisomatales*	0.70	95.70	2.69	470	5.78	-
C14-07	B	f_*Desulfurivibrionaceae*	0.63	98.80	2.35	287	2.52	-
C14-12	B	p_*Zixibacteria*	0.46	96.70	1.20	242	3.06	-
C14-18	A	g_*Ca.* Methanovorans	0.44	95.01	0.98	263	1.88	-
C14-11	B	f_*Spirochaetaceae*	0.43	97.20	2.00	344	3.68	-
C14-24	B	f_*Trueperaceae*	0.40	84.95	2.97	666	3.26	696
C14-20	A	f_*Thorarchaeaceae*	0.39	90.18	3.27	703	4.03	1556
C14-25	B	o_*Bacteroidales*	0.36	83.63	3.83	1235	6.00	-
C14-23	B	g_*Lutibacter*	0.34	85.01	3.24	674	2.49	-
C14-22	B	g_*Sedimenticola*	0.34	85.63	1.39	663	3.03	-
C14-26	B	c_*Anaerolineae*	0.33	80.49	0.48	1021	4.22	-

* The lowest taxon with a published name according the GTDB classification.

## Data Availability

Data are contained within the article and supplementary materials. All the sequencing data are deposited in NCBI BioProject PRJNA940400. The MAGs Ac-cession Numbers are JARGFH000000000—JARGGJ000000000.

## Supplementary Materials

The following supporting information can be downloaded at: https://www.mdpi.com/article/10.3390/microorganisms12071349/s1, Table S1: Functional genes; Table S2: qPCR; Table S3: Alfa diversity; Figure S1: Phylogenetic analysis of the ANME MAGs. Refs. [76,77,78,79] can be found in Supplementary Materials.
