# Supplementary material for: Diversity, Methane Oxidation Activity, and Metabolic Potential of Microbial Communities in Terrestrial Mud Volcanos of the Taman Peninsula
Source: Microorganisms. 2024 Jul 1;12(7):1349. doi: 10.3390/microorganisms12071349 (PMC11279179; doi:10.3390/microorganisms12071349)
Supplement: Supplementary file 1 [file microorganisms-12-01349-s001.zip › Supplementary Figure S1_ANME tree.pdf]

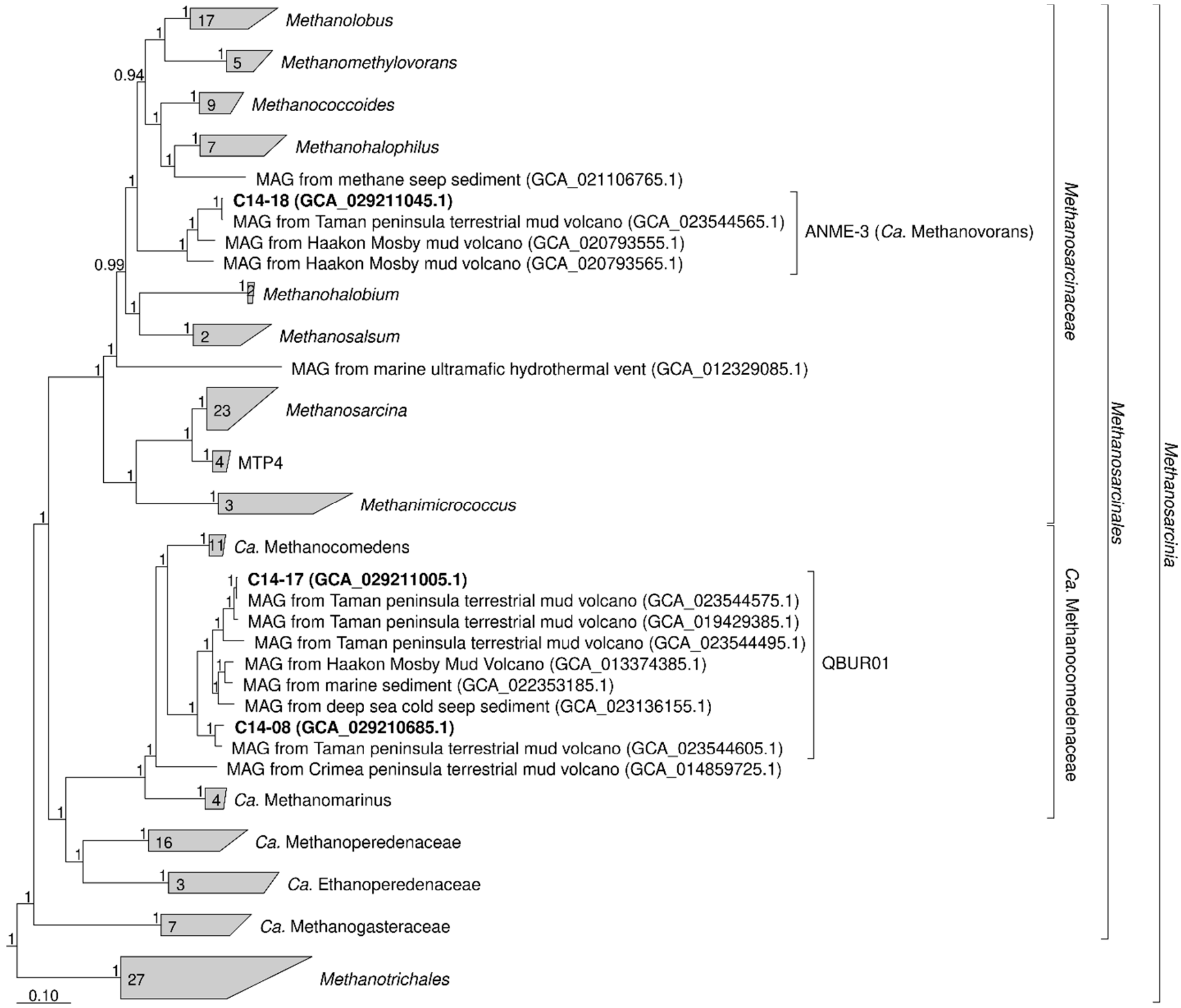

Figure S1. Phylogenomic placement of ANME MAGs based on concatenated partial amino acid sequences of 122 archaeal single copy conserved marker genes with taxonomic designations according to the GTDB. The tree was built using the IQ-TREE 2 program [Minh et al., 2020] with fast model selection via ModelFinder [Kalyaanamoorthy et al., 2017] and ultrafast bootstrap approximation [Minh et al., 2013], as well as an approximate likelihood-ratio test for branches [Anisimova and Gascuel, 2006]. Bootstrap consensus tree is shown with values placed at the nodes. Bar, 0.1 changes per position.

References

[76] Minh, B.Q.; Schmidt, H.A.; Chernomor, O.; Schrempf, D.; Woodhams, M.D.; von Haeseler, A.; Lanfear, R. IQ-TREE 2: New models and efficient methods for phylogenetic inference in the genomic era. Mol. Biol. Evol. 2020, 37, 1530–1534.

[77] Kalyaanamoorthy, S.; Minh, B.Q.; Wong, T.; von Haeseler, A.; Jermiin, L.S. ModelFinder: Fast model selection for accurate phylogenetic estimates. Nat. Methods 2017, 14, 587–589.

[78] Minh, B.Q.; Nguyen, M.A.; von Haeseler, A. Ultrafast approximation for phylogenetic bootstrap. Mol. Biol. Evol. 2013, 30, 1188–1195.

[79] Anisimova, M.; Gascuel, O. Approximate likelihood-ratio test for branches: A fast, accurate, and powerful alternative. Syst. Biol. 2006, 55, 539–552.
